# Liprin‐α1 contributes to oncogenic MAPK signaling by counteracting ERK activity

**DOI:** 10.1002/1878-0261.13593

**Published:** 2024-01-24

**Authors:** Henna Pehkonen, Artemis Filippou, Juho Väänänen, Iida Lindfors, Mira Vänttinen, Philipp Ianevski, Anne Mäkelä, Pauliina Munne, Juha Klefström, Sanna Toppila‐Salmi, Reidar Grénman, Jaana Hagström, Antti A. Mäkitie, Piia‐Riitta Karhemo, Outi Monni

**Affiliations:** ^1^ Applied Tumor Genomics Research Program, Faculty of Medicine University of Helsinki Finland; ^2^ Institute for Molecular Medicine Finland (FIMM) University of Helsinki Finland; ^3^ Finnish Cancer Institute, FICAN South Helsinki University Hospital & Translational Cancer Medicine, Medical Faculty University of Helsinki Finland; ^4^ iCAN Digital Precision Cancer Medicine Flagship Helsinki Finland; ^5^ Skin and Allergy Hospital Helsinki University Hospital and University of Helsinki Finland; ^6^ Department of Otorhinolaryngology, Kuopio University Hospital and School of Medicine, Institute of Clinical Medicine University of Eastern Finland Kuopio Finland; ^7^ Department of Otorhinolaryngology‐Head and Neck Surgery University of Turku and Turku University Hospital Finland; ^8^ Department of Pathology University of Helsinki and Helsinki University Hospital Finland; ^9^ Institute of Dentistry University of Turku Finland; ^10^ Department of Otorhinolaryngology‐Head and Neck Surgery, Research Program in Systems Oncology University of Helsinki and Helsinki University Hospital Finland; ^11^ Department of Oncology, Faculty of Medicine University of Helsinki Finland

**Keywords:** drug screen, head and neck squamous cell carcinoma, liprin‐α1, MEK/ERK inhibitor, MEK/ERK signaling pathway, RAS

## Abstract

PTPRF interacting protein alpha 1 (*PPFIA1*) encodes for liprin‐α1, a member of the leukocyte common antigen–related protein tyrosine phosphatase (LAR‐RPTPs)‐interacting protein family. Liprin‐α1 localizes to adhesive and invasive structures in the periphery of cancer cells, where it modulates migration and invasion in head and neck squamous cell carcinoma (HNSCC) and breast cancer. To study the possible role of liprin‐α1 in anticancer drug responses, we screened a library of oncology compounds in cell lines with high endogenous *PPFIA1* expression. The compounds with the highest differential responses between high *PPFIA1*‐expressing and silenced cells across cell lines were inhibitors targeting mitogen‐activated protein kinase kinase (MEK)/extracellular signal‐regulated kinases (ERK) signaling. KRAS proto‐oncogene, GTPase (*KRAS*)‐mutated MDA‐MB‐231 cells were more resistant to trametinib upon *PPFIA1* knockdown compared with control cells. In contrast, liprin‐α1‐depleted HNSCC cells with low RAS activity showed a context‐dependent response to MEK/ERK inhibitors. Importantly, we showed that liprin‐α1 depletion leads to increased p‐ERK1/2 levels in all our studied cell lines independent of *KRAS* mutational status, suggesting a role of liprin‐α1 in the regulation of MAPK oncogenic signaling. Furthermore, liprin‐α1 depletion led to more pronounced redistribution of RAS proteins to the cell membrane. Our data suggest that liprin‐α1 is an important contributor to oncogenic RAS/MAPK signaling, and the status of liprin‐α1 may assist in predicting drug responses in cancer cells in a context‐dependent manner.

## Graphical abstract

Liprin‐α1 is a scaffold protein involved in cancer cell adhesion and invasion. We show that liprin‐α1 depletion significantly alters the response of HNSCC and breast cancer cells to MEK/ERK inhibitors and leads to increased p‐ERK levels and redistribution of RAS proteins to the cell membrane. Thus, liprin‐α1 is an important contributor to oncogenic RAS/MAPK signaling and a potential drug response indicator for MEK/ERK inhibitors.

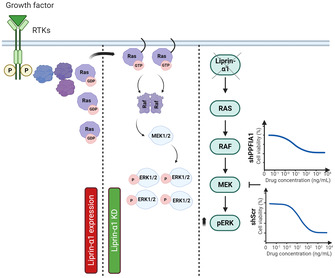

**Abbreviations**
DSRT: drug sensitivity and resistance testing
DSS: drug sensitivity score
EGFR: epidermal growth factor receptor
ERK: extracellular signal‐regulated kinases
HNSCC: head and neck squamous cell carcinoma
IAP: inhibitor of apoptosis proteins
*KRAS*
: Kirsten rat sarcoma proto‐oncogene, GTPase
LAR‐RPTP: leukocyte common antigen‐related protein tyrosine phosphatase
MEK: mitogen‐activated protein kinase
MPAS: MAPK pathway activity score
mTOR/PI3K: mammalian target of rapamycin/phosphatidylinositol‐3‐kinase
PPFIA1: PTPRF interacting protein alpha 1
shRNA: short hairpin RNA

## 1 Introduction

Liprin‐α1 is a member of the liprin (LAR [leukocyte common antigen related]‐interacting protein) family [1, 2]. Liprin‐α1 is encoded by *PPFIA1*, located at the 11q13 amplification region, which is common in cancer, including head and neck squamous cell carcinoma (HNSCC) and breast cancer [3, 4, 5, 6]. Liprin‐α1 functions in adhesion‐related cell signaling, extracellular matrix degradation, and cell invasion in cancer cells [7, 8, 9, 10]. Liprin‐α1 plays a role in the invadosome function and organization including their maturation [7, 8, 11], in cell spreading and lamellipodia function [8, 12, 13, 14], as well as in β1‐integrin recycling and adhesion [1, 14, 15, 16]. Liprin‐α1 is also a component of plasma membrane–associated platforms [17, 18]. Liprin‐α1 is an important player in the regulation of metastatic burden *in vivo* by promoting protrusive activity at the leading edge of the migrating breast cancer cells [19]. We have recently shown that *PPFIA1* knockdown leads to upregulation of transmembrane protein CD82 [9], an important suppressor of metastasis, as well as a modulator of several pathways important in cancer cell signaling [20, 21].

Previous data have shown the potential of high‐throughput screening in predicting the association of common genetic aberrations or biomarker expression to drug response [22, 23, 24, 25]. The ERK protein kinases of the MAPK family convey mitogen‐ or growth factor‐induced signals by a phosphorylation cascade transduced by RAS GTPases [26]. The ERK proteins have a variety of substrates via which they regulate distinct molecular processes and dysregulation of the pathway commonly occurs by mutations upstream of ERKs, for example, in RAS [27]. Typically, oncogenic mutations in RAS cause the protein to remain in a GTP‐bound activated state, thereby leading to a constant activation of downstream targets [27]. Mitogen‐activated protein kinase is considered a main target for inhibition of ERK pathway, since activation of MEK is sufficient for ERK and subsequent downsteam pathway activation [28]. Trametinib is a competitive inhibitor of MEK1/2, approved by the FDA for the treatment of unresectable melanoma [29] and non‐small cell lung cancer with BRAFV600 mutation [30]. Tumor cells often evade MEK inhibition via rewiring and activation of MAPK, indicating the dependence of many cancers on this pathway for survival and proliferation [31].

To explore whether liprin‐α1 is a potential drug response biomarker, we carried out a drug sensitivity and resistance testing (DSRT) utilizing FDA approved and investigational compounds for cell lines with high endogenous *PPFIA1* expression, as well as their liprin‐α1‐depleted counterparts. Our findings suggest that liprin‐α1 is a potential drug response indicator of anticancer compounds including several MEK inhibitors. Breast cancer and HNSCC liprin‐α1 knockdown cell lines showed differential response to MEK/ERK inhibition, which might be due to the *KRAS* mutational status or crosstalk between MEK/ERK and PI3K/mTOR signaling pathways. Altogether, we show that liprin‐α1 counteracts p‐ERK1/2 protein levels and RAS activity and thus, has regulatory functions in oncogenic MAPK signaling.

## 2 Materials and methods

### 2.1 Cell lines and reagents

Breast carcinoma cell lines MDA‐MB‐231 (CVCL_0062) and BT‐474 (CVCL_0179) were obtained from ATCC (Manassas, VA, USA) and HNSCC cell lines UT‐SCC‐24A (CVCL_7826), UT‐SCC‐42A (CVCL_7847), UT‐SCC‐42B (CVCL_7848), and UT‐SCC‐95 (CVCL_A7EQ) were obtained from R. Grénman (Department of Otorhinolaryngology‐Head and Neck Surgery, University of Turku and Turku University Hospital, Finland). All original cell lines were authenticated at the Institute for Molecular Medicine Finland (FIMM). All utilized cell lines in this study have been tested negative for mycoplasma contamination with MycoAlert Detection Kit (Lonza, Basel, Switzerland). Breast carcinoma and HNSCC cell lines were cultured in DMEM (Lonza) supplemented with 10% fetal bovine serum (FBS) 100 U·mL^−1^ penicillin/streptomycin (Lonza), 2 mm l‐glutamin (Lonza), and 0.1 mm non‐essential amino acids (NEAA) (Lonza). Antibodies used were rabbit polyclonal liprin‐α1 (Proteintech, Manchester, UK), mouse monoclonal α‐tubulin (Sigma‐Aldrich, Saint Louis, MO, USA), p44/42 MAPK (ERK1/2) Antibody (#9102), phospho‐p44/42 MAPK (pERK1/2) (Thr202/Tyr204) rabbit mAb (#4377), rabbit monoclonal p‐AKT (Ser473) (#4060), p‐rS6 rabbit mAb (Ser 235/236) (#4858), p‐MEK1/2 (Ser217/221) (41G9) rabbit mAb (#9154), MEK1/2 (#8727) rabbit mAb (Cell Signaling, Danvers, MA, USA), mouse mAb RAF1 (E10, sc7267) (Santa Cruz, Dallas, TX, USA), rabbit mAb RAF1 (Abcam, Cambridge, UK), mouse monoclonal vinculin (Sigma‐Aldrich), and mouse monoclonal GAPDH (Europa Bioproducts, Wicken, UK). Specifically for pan‐RAS, rabbit monoclonal ab52939 RAS (EP1125Y) was used for IF, and mouse monoclonal pan‐RAS (Ab‐1, DWP; Calbiochem, Darmstadt, Germany) or monoclonal anti‐pan‐RAS antibody (AESA02; Cytoskeleton, Denver, CO, USA) for WB and RAS activation assay. Trametinib (Selleckchem, Houston, TX, USA) was used to validate the results from the drug screen. Dimethyl sulfoxide (DMSO) was used as a control.

### 2.2 Lentiviral transduction and constructs

To knockdown *PPFIA1*, shRNA constructs were purchased from TRC1 and TRC2 library (Sigma‐Aldrich). Altogether, three shRNA constructs for liprin‐α1 (TRCN0000342514, TRCN0000380944, and TRCN0000002969) were utilized for stable transductions of the cell lines. Throughout the article, TRCN0000002969 corresponds to shPPFIA_1, TRCN0000342514 to shPPFIA1_2, and TRCN0000380944 to shPPFIA1_3, respectively. Construct shPPFIA1_1 was selected for the initial large‐scale drug screen, because it most effectively knocks down *PPFIA1* expression [7, 9]. For the rest of the experiments, at least two out of three different shRNA constructs were utilized. Scramble shRNA (shScramble, SHC002) was used as a control. Open reading frame (ORF) for PPFIA1 (clone ID 4794300) was ordered from the ORFeome Collection (Open Biosystems, Pittsburgh, PA, USA) and cloned from donor pENTR221 vector into lentiviral destination expression vector pLenti6/V5 DEST (Invitrogen, Carlsbad, CA, USA) using Gateway cloning system. Empty pLenti6/V5 DEST vector was used as a control. Gateway cloning was done at the Genome Biology Unit, University of Helsinki, Finland. Generation of viral particles was done at the Biomedicum Functional Genomics Unit (University of Helsinki, Finland), and generation of transduced cell lines has been described earlier [7]. Briefly, polybrene (200 μg·mL^−1^) was used in transduction of the cells with the viral particles. Media was changed after incubation of the cells with polybrene and viral particles for 4 h. After 72‐h incubation, the cells were selected using 1 μg·mL^−1^ of puromycin (Sigma‐Aldrich).

### 2.3 Drug sensitivity and resistance testing screen

Drug sensitivity and resistance testing screen of 527 anticancer drugs was performed for the MDA‐MB‐231 cells at the Institute for Molecular Medicine Finland (FIMM, University of Helsinki, Finland), as previously described [25]. Targeted screen with 23 compounds, selected based on the results from MDA‐MB‐231, was performed for UT‐SCC‐24A, UT‐SCC‐42A, UT‐SCC‐95, and BT‐474 cell lines. Briefly, all compounds were tested over a 10 000‐fold concentration range in five different concentrations in 384‐well plates to generate quantitative and reliable dose–response data. Drugs were dispensed on plates by acoustic liquid handler (Echo 550; Labcyte, Lakeview, IN, USA) and dissolved in 5 μL of media. The cells were plated at a density of 1000 cells/well (MDA‐MB‐231) and 1250 cells/well (all the other cell lines) in 20 μL volume by MultiDrop Combi peristaltic dispenser (Thermo Scientific, Waltham, MA, USA) and incubated at +37 °C and 5% CO_2_ for 72 h. After 72 h, cell viability was measured by CellTiter‐Glo luminescent assay (Promega, Madison, WI, USA). In addition, cytotoxicity was measured by luminescence‐based CellTox Green (Promega) assay, which quantifies changes in membrane integrity that occurs due to cell death. Dimethyl sulfoxide and benzethonium chloride (100 μmol·L^−1^) were used as a negative and positive control, respectively. The drug sensitivity was measured by drug sensitivity score (DSS), which integrates the efficacy of drug concentration, the half maximal inhibitory concentration (IC50), the half maximal effective concentration (EC50), and the maximal inhibition [32]. Analysis of the drug screen data was carried out in r studio software (Boston, MA, USA; r 3.6.3). graphpad prism software (Dotmatics, San Diego, CA, USA; version 9.2.0) was used for data visualization. Drug screening experiments for MDA‐MB‐231 cell line were carried out using transduced cells with a shScramble and one shPPFIA1 construct (shPPFIA1_1), while for UT‐SCC‐42A, UT‐SCC‐24A and BT‐474, cells were stably transduced with shScramble and two different knockdown constructs (shPPFIA1_1 and shPPFIA1_2). An average of DSS of the two different constructs was calculated for these cells. UT‐SCC‐95 cell line was transduced with *PPFIA1* ORF construct. Empty vector was used as a control. To study the expression of selected proteins after trametinib treatment, UT‐SCC‐42A and MDA‐MB‐231 shScramble and shPPFIA1 cells were seeded into 100‐mm plates (Corning, New York, NY, USA) and treated with DMSO (control) and different trametinib concentrations (1, 10, and 100 nm). Cells were incubated for 48 h after which the cell lysates were collected for western blot analysis.

### 2.4 Trypan blue cell viability assay

MDA‐MB‐231 cells (shScramble and shPPFIA1) were seeded on the cell culture plates overnight and treated with DMSO (negative control) and 100 nm trametinib for 24 h followed by addition of trypan blue. Three replicate experiments in all conditions were performed. Cells with nonintact membrane that take up trypan blue in their cytoplasm (nonviable) were calculated and Student's *t*‐test was used to count statistical significance.

### 2.5 Western blot

Western blot was performed as described earlier [7]. Briefly, cells were lysed with RIPA lysis buffer (Sigma‐Aldrich), which was supplemented with protease and phosphatase inhibitors (Roche, Basel, Switzerland). Protein concentration from the lysates was measured by BCA protein assay kit (Pierce, Waltham, MA, USA, and/or Bio‐Rad, Hercules, CA, USA). Proteins were transferred to polyvinylidene fluoride (PVDF) membrane using Trans‐Blot Turbo equipment (Bio‐Rad). Blocking of the membrane was performed with 5% milk or BSA in Tris‐buffered saline and Tween (TBST). Primary antibody dilution was 1 : 1000 in 1% milk or BSA in TBST and the membrane was incubated overnight. Secondary antibody dilution was 1 : 10 000–20 000 in 1% milk or BSA in TBST. Membranes were washed with TBST washing buffer. Detection reagents for chemiluminescence were from Merck‐Millipore (Burlington, MA, USA). Chemidoc (Bio‐Rad) and adobe photoshop cc (Adobe Inc., San Jose, CA, USA) were used to visualize the results. Quantification of western blots were carried out by measuring the intensity of each band and subtracting the background by using the chemidoc software (Bio‐Rad). Intensities of experimental blots were then quantified against loading controls to get the fold changes for each measurement. Error bars were counted as a standard deviation and two‐tailed Student's *t*‐test was used for the statistical analysis.

### 2.6 Immunofluoresence

Immunofluoresence was performed as previously described [7, 9]. Briefly, cells were fixed with 4% PFA in PBS. Cells were washed with PBS and incubated in 0.1% Triton X‐100 in PBS for 5 min. Cells were washed again with PBS followed by incubation of the cells with 0.12% glycine in PBS for 10 min. Cells were then washed with PBS and incubated in blocking solution 3% BSA in PBS for 30 min. Cells were incubated with primary antibody for 1 h in room temperature (RT) followed by washes with PBS. After incubation of the cells RT for 1 h with secondary antibody, the cells were washed with PBS and MQ water. Finally, the cells were placed in coverslips in mounting medium Mowiol with DAPCO and DAPI to stain the nucleus.

### 2.7 Microscopy

Immunofluorescence stainings were imaged with confocal microscope Zeiss LSM 780/880 (Zeiss, Oberkochen, Germany; 63×). zen software (Trumbull, CT, USA), adobe photoshop cc, adobe illustrator cc (Adobe Inc.) and imagej (National Institutes of Health, Bethesda, MD, USA) were used for image processing. Quantification of immunofluorescence images was performed with the imagej software. To quantify the shScr and shPPFIA1 images, same settings were used with shScr and shPPFIA1 in individual experiments. A minimum of 70 cells were analyzed three times from two or three different experiments. The intensities were thresholded in imagej, and error bars were calculated by standard deviation of the mean. For p‐ERK, the intensity values for each image were calculated and divided by amount of cells, which were counted from DAPI stainings. For membrane localization of RAS, cell membrane intensities were thresholded and divided by the area of the cells. Student's two‐tailed *t*‐test was used for statistical analysis.

### 2.8 RAS activation assay

Cells were counted, seeded into 14‐cm‐diameter culture dishes and maintained with 10% serum‐containing medium until reaching 70% confluent. After 15 h of serum deprivation, cells were washed with ice‐cold PBS and lysed by scraping on ice with 400 μL of high magnesium‐containing buffer (50 mm Tris pH 7.5, 10 mm MgCl_2_, 0.5 m NaCl and 2% Igepal) supplemented with protease and phosphatase inhibitors (Roche, cocktail). The total activated RAS in each cell line was assessed using RAS Activation Assay Biochem Kit (Cytoskeleton) according to the manufacturer's instructions. Briefly, lysates were clarified by centrifugation at 10 000 ***g***, 4 °C for 1 min. Clarified aliquots from the lysates were stored separately for protein quantification and western blot of total RAS. Following protein concentration determination using Pierce BCA protein Assay Kit, samples from cell extracts were equalized with ice‐cold lysis buffer to achieve identical protein concentrations. Cell lysates were equalized to contain 300 μg of total protein per sample and Raf‐GST agarose beads (30 μL) were added to each reaction. The reaction mixtures were gently rotated at 4 °C for 1 h. After microcentrifugation at 5000 ***g*** at 4 °C for 1 min, supernatants were removed, and the beads were washed quickly with magnesium‐containing wash buffer. Agarose beads were pelleted by centrifugation at 5000 ***g*** at 4 °C for 3 min, and supernatant was carefully removed. The beads were resuspended in 20 μL of 2× Laemmli buffer with β‐mercaptoethanol and boiled for 2 min. Inputs and immunoprecipitation samples were analyzed separately by SDS/PAGE and western blot analysis. Membranes were blocked with 5% nonfat dry milk in TBST for 30 min at RT with constant agitation, according to the manufacturer's instructions. The membrane with pulled‐down samples was incubated with 1 : 250 dilution of anti‐pan RAS antibody provided with the kit overnight at 4 °C with constant agitation. The following day the blot was washed three times in TBST and incubated with horseradish peroxidase‐conjugated secondary antibody at a 1 : 20 000 dilution in 1% nonfat milk in TBST. Standard chemiluminescence western blotting protocol was used to detect the immunoreactive bands.

### 2.9 MAPK pathway activity score

MAPK pathway activity score (MPAS) was calculated for each cell line as described previously [33]. *Z*‐scores were computed from normalized gene expression values of 10 MAPK pathway genes (*CCND1*, *DUSP4*, *DUSP6*, *EPHA2*, *EPHA4*, *ETV4*, *ETV5*, *PHLDA1*, *SPRY2*, and *SPRY4*). The *z*‐scores were then summed for each sample, and the total sum was divided by the square root of the number of genes in the gene set (*n* = 10).

### 2.10 Microarray data analysis

The array expression data [7] were analyzed with ThermoFisher Scientific Transcriptome Analysis Console (TAC v4.0.3). Data were normalized using Robust Multichip Average (RMA), and probes were collapsed to genes using extended gene‐level summarization. One control and two overexpressing replicates were used in the analysis.

### 2.11 RNA sequencing data analysis

RNA‐seq data [7] were preprocessed using qualimap, fastqc, trimmomatic, and the star aligner (GitHub Inc., San Francisco, CA, USA; v2.7.11a). For the reference genome and gene annotations, GENCODE Release 44 (GRCh38.p14) files were used. Sample clustering was inspected by PCA plotting, and outliers were removed. Differential expression analysis was performed by the deseq2 package in r (Boston, MA, USA; v4.3.1). For heatmaps, the normalized expression values were further processed by performing variance‐stabilizing transformation before plotting. In MDA‐MB‐231 analysis, three shScramble and three shPPFIA1 replicates were used, and in UT‐SCC‐42A analysis, three shScramble and two shPPFIA1 replicates were used.

## 3 Results

### 3.1 *KRAS*‐mutated MDA‐MB‐231 cells become more resistant to MEK inhibitors upon liprin‐α1 depletion

**Fig. 1:**
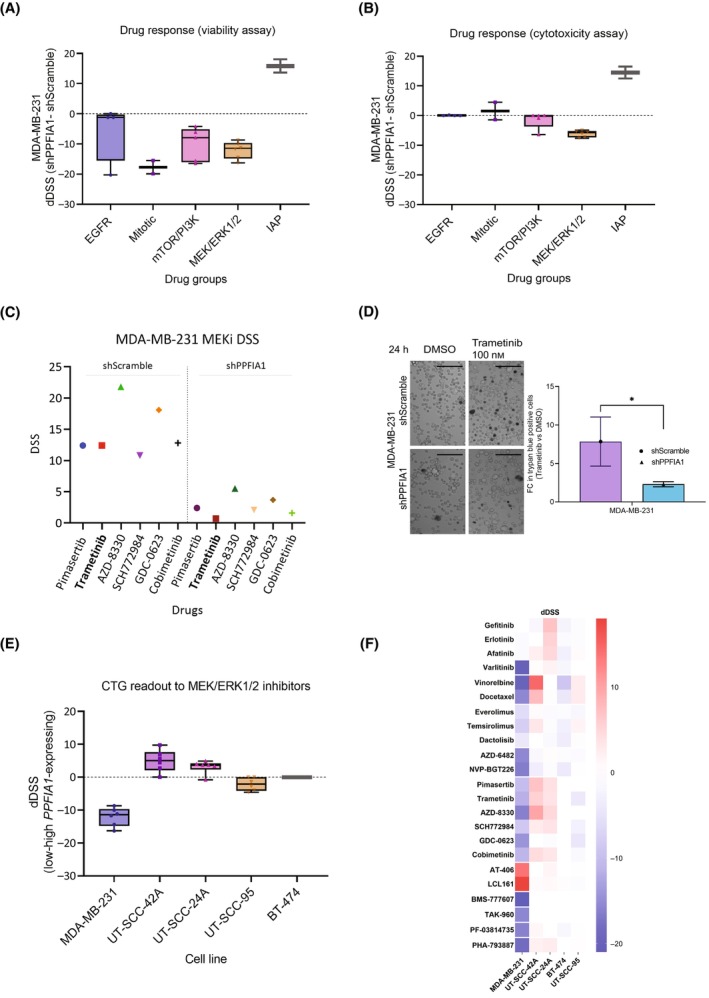
Comparison of drug responses between shPPFIA1 and shScramble cells. (A, B) Box plots visualizing the difference in drug responses between MDA‐MB‐231 shPPFIA1 (knockdown) and shScramble (control) cells. The drug screen consisted of a total of 527 compounds and the figure shows responses of the cells to selected functional drug classes, including inhibitors for EGFR (epidermal growth factor receptor; *n* = 4), mitotic inhibitors (*n* = 2), inhibitors for mTOR/PI3K (The mammalian target of rapamycin/Phosphatidylinositol‐3‐kinase signaling; *n* = 5), MEK/ERK [mitogen‐activated protein kinase kinase (MEK)/extracellular signal‐regulated kinases (ERK) signaling (*n* = 6)], and IAP (inhibitor of apoptosis proteins; *n* = 2). The drug sensitivity was measured by drug sensitivity score (DSS), which integrates the efficacy of drug concentration, the half maximal inhibitory concentration (IC50), the half maximal effective concentration (EC50) and the maximal inhibition [32]. The difference in drug response of specific drug classes between shPPFIA1 and shScramble cells was calculated as the differential drug sensitivity score (dDSS) by subtracting shScramble DSS from shPPFIA1 DSS, as measured by CellTiter‐Glo cell viability assay (A) and CellTox Green cell cytotoxicity assay (B). (C) DSS for MEK/ERK inhibitor treated MDA‐MB‐231 shScramble and shPPFIA1 cells showing clear difference in drug responses between shScramble and shPPFIA1 cells. Trametinib is shown in bold, because it was used to validate the drug screen results in MDA‐MB‐231 shScramble and shPPFIA1 cells. Each condition was screened once in a high throughput manner. (D) MDA‐MB‐231 shScramble cells with high *PPFIA1* expression were more sensitive to 100 nm trametinib, as compared to *PPFIA1* knockdown cells (shPPFIA1), when measured by trypan blue assay. Fold change (FC) of trypan blue positive cells in trametinib versus DMSO‐treated (negative control) conditions was calculated for both the shScramble and shPPFIA1 cells. Trametinib was significantly more effective for shScramble cells. Asterisk (*) indicates statistical significance (*P* < 0.05). Scale bar is 0.07 mm. Error bars indicate the standard deviation of the mean and each condition (shScramble and shPPFIA1) was measured in three replicates (*n* = 3). Student's *t*‐test was used to calculate the statistical significance. (E) Box plots showing differences in drug sensitivity scores (dDSS) between MEK/ERK inhibitor treated MDA‐MB‐231, UT‐SCC‐42A, UT‐SCC‐24A, and BT‐474 cell lines (shPPFIA1 – shScramble; *n* = 6 for each cell line). dDSS of UT‐SCC‐95 cell line ectopically expressing liprin‐α1 was compared to the empty vector (control‐overexpressing cells; *n* = 6). The drug responses presented in the box plots were measured by using a cell viability assay (CTG; CellTiter‐Glo). (F) Heatmap illustrating the differences in DSS between shScramble and shPPFIA1 in MDA‐MB‐231, UT‐SCC‐42A, UT‐SCC‐24A, and BT‐474 cells. For UT‐SCC‐95 cells, heatmap shows the dDSS between cells with ectopic *PPFIA1* expression and control. Drug sensitivity scores are color‐coded, ranging from positive response (red) to negative response (blue) in shScramble and shPPFIA1 cells as well as in *PPFIA1* overexpressing and control cells.

As *PPFIA1* gene is amplified in about 15% of breast cancers [34] and liprin‐α1 protein plays a role in cancer cell migration and invasion [7, 19], we sought to explore its possible role as a biomarker of drug response. To investigate the druggable vulnerabilities of MDA‐MB‐*231 PPFIA1‐*expressing (shScramble) or knockdown cells (shPPFIA1), we carried out a systematic DSRT screen of 527 FDA‐approved and investigational compounds (Table S1A). MDA‐MB‐231 breast cancer cell line was selected for the screen, since *PPFIA1* knockdown has been previously shown to induce inhibition of its invasive phenotype [8]. *PPFIA1* knockdown decreased the sensitivity of MDA‐MB‐231 cells to mitotic, mTOR/PI3K and MEK/ERK inhibitors, as measured by viability assay (Fig. 1A; Table S1). On the contrary, *PPFIA1* knockdown sensitized MDA‐MB‐231 cells to inhibitors of apoptosis (IAP) supported by both cell viability and cytotoxicity assays (Fig. 1A,B; Table S1A). Interestingly, MDA‐MB‐231 shPPFIA1 cells were more resistant to several MEK/ERK inhibitors (pimasertib, trametinib, SCH772984, AZD‐8330, GDC‐0623, and cobimetinib) as observed by both the cell viability (DSS ≤ 5.5 vs DSS > 10) and cytotoxicity assays (Fig. 1C; Table S1A). To further validate the effect of MEK inhibitor response, we treated the cells with 100 nm trametinib followed by quantification of the cells by trypan blue assay. The response was more effective in shScramble cells compared with *PPFIA1* knockdown cells (Fig. 1D). These results suggest that *PPFIA1* expression may be an indicator for response to several anticancer drugs related to oncogenic signaling in MDA‐MB‐231.

### 3.2 Liprin‐α1 modulates MEK/ERK inhibitor response in HNSCC cell lines

To further explore the impact of liprin‐α1 expression to targeted inhibition of oncology compounds, we carried out a drug screen of 23 compounds, which have shown the biggest differences in drug response (dDSS) from the original screen. A targeted drug screen was carried out for UT‐SCC‐42A and UT‐SCC‐24A HNSCC cells (shScramble and shPPFIA1), which have endogenously high expression of liprin‐α1 and they lack *KRAS/BRAF* mutations [35]. Interestingly, *PPFIA1* knockdown sensitized UT‐SCC‐42A and UT‐SCC‐24A cells to several MEK/ERK inhibitors, contrary to MDA‐MB‐231 cells (Fig. 1E,F; Table S1A–C). A heatmap highlights the differences in DSS between shScramble and *PPFIA1* knockdown in all the studied cell lines (Fig. 1F) with the group of MEK/ERKi showing substantial difference across cell lines between control and knockdown cells. Similarly, mitotic inhibitors vinorelbine and docetaxel showed opposite effects in UT‐SCC‐42A and MDA‐MB‐231 cells (Fig. 1F; Table S1A,B, Fig. S1A). The differential drug responses are likely indicative of variation in upstream or downstream pathway activation upon liprin‐α1 silencing in MDA‐MB‐231 and UT‐SCC cells. To further explore the role of liprin‐α1 in either sensitizing or making cells more resistant to MEK inhibitors, we studied BT‐474 breast cancer cell line, which is resistant to MEK inhibitors, as well as UT‐SCC‐95 HNSCC cell line in which we ectotopically expressed liprin‐α1. Neither of these cell lines have *KRAS*/*BRAF* mutations. Liprin‐α1 depletion did not make BT‐474 more sensitive to MEK inhibitors (Fig. 1E; Table S1D). *PPFIA1*‐overexpressing UT‐SCC‐95 cells, on the other hand, became more sensitive to MEK inhibitors (Table S1E) suggesting that liprin‐α1 modulates MEK inhibitor drug responses but the effect is context‐dependent (Fig. 1E,F; Tables S2–S24).

### 3.3 Liprin‐α1 knockdown results in increased pERK levels

**Fig. 2:**
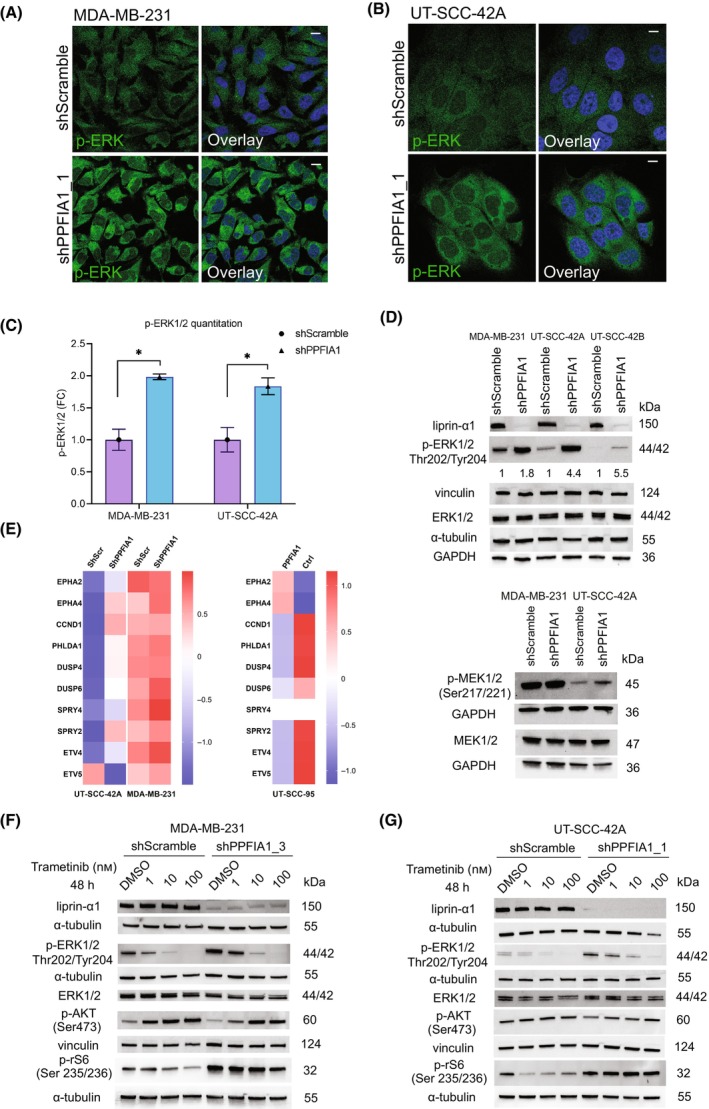
Effect of liprin‐α1 knockdown to ERK phosphorylation and to oncogenic signaling. (A) Localization and expression of p‐ERK1/2 (Thr202/Tyr204) in MDA‐MB‐231 shScramble and shPPFIA1_1 (construct #69) cells as visualized by immunofluorescence microscopy. Scale bar is 10 μm. (B) Localization and expression of p‐ERK1/2 (Thr202/Tyr204) in UT‐SCC‐42A shScramble and shPPFIA1_1 (construct #69) cells as visualized by immunofluorescence microscopy. Scale bar is 10 μm. (C) Quantification of p‐ERK1/2 staining in A and B is shown in bar chart as a fold change (FC) between shScramble and shPPFIA1 cells and asterisk (*) means statistical significance (*P* < 0.05). Student's two‐tailed *t*‐test was used to calculate the statistical significance. Error bar indicates the standard deviation of the mean. A minimum of 70 individual cells were quantitated three times from two (shScramble) or three (shPPFIA1) different experiments for both of the cell lines and representative images are shown for MDA‐MB‐231 cell line in 2A and for UT‐SCC‐42A in 2B. (D) Western blot showing protein levels of liprin‐α1, p‐ERK1/2 (Thr202/Tyr204), ERK1/2, MEK1/2 and p‐MEK1/2 (Ser217/221) in a panel of studied shScramble and shPPFIA1 cells. GAPDH, α‐tubulin and vinculin were used as the loading controls. The intensity of p‐ERK1/2 bands was quantified for individual cell lines and the values are described under the blot. In addition, western blot for UT‐SCC‐42A and UT‐SCC‐42B was performed with two different constructs for p‐ERK (Fig. S2D). (E) Heatmap showing MAPK pathway activity score in MDA‐MB‐231 and UT‐SCC‐42A shScr and shPPFIA1 cells, and in UT‐SCC‐95 control and *PPFIA1* overexpressing cells. For MDA‐MB‐231 and UT‐SCC‐42A cells, *z*‐scores were computed from previously published RNAseq data [9]. For UT‐SCC‐95 cell line, *z*‐scores were computed from the RMA‐normalized expression values from microarrays [7]. The *EPHA4* gene had two probe sets mapping to the same gene, so the mean expression value was used as a basis for the *z*‐score. Finally, the mean *z*‐scores from different constructs were calculated. For MDA‐MB‐231 cell line, three replicates of shScr and shPPFIA1 cells were included into the analysis whereas for UT‐SCC‐42A cell line, three replicates from shScr cells and two replicates from shPPFIA1 cells were analyzed. For UT‐SCC‐95, one control and two overexpressing samples were included into the analysis. (F, G) Western blot analysis from MDA‐MB‐231 (F) and UT‐SCC‐42A (G) cells treated 48 h with trametinib. Protein levels of liprin‐α1, ERK1/2, p‐ERK1/2 (T202/Y204), p‐AKT (Ser473) and p‐rS6 (Ser235/236) proteins are shown for shScramble and shPPFIA1 cells. DMSO‐treated cells were used as a negative control for trametinib treatment, whereas α‐tubulin and vinculin served as loading controls. Figure S3C,D shows quantification of p‐ERK1/2 immunoblot results calculated from four experiments for MDA‐MB‐231 and five experiments for UT‐SCC‐42A. Liprin‐α1, p‐AKT and p‐rS6 western blot experiments were performed twice for each cell line and each condition (Fig. 2F,G; Fig. S3A,B).

According to our previous data and gene expression analysis, *PPFIA1* knockdown leads to increased mRNA expression of *MAPK1* and *MAP2K1* genes encoding for ERK and MEK proteins, respectively [9]. However, to our knowledge, no data on the role of liprin‐α1 in the regulation of MEK/ERK activation are previously reported. This led us to study the effects of liprin‐α1 to ERK signaling. *PPFIA1* knockdown resulted in increased cytoplasmic levels of p‐ERK in MDA‐MB‐231 and UT‐SCC‐42A cells (Fig. 2A–C). Furthermore, *PPFIA1* knockdown led to increased ERK activity in all our tested cell lines, defined by immunoblotting of phosphorylated ERK1/2 at residues Thr202/Tyr204 (Fig. 2D). As expected, basal p‐ERK activity was higher in MDA‐MB‐231 cells as compared to nonmutated UT‐SCC‐42A cells due to its KRAS/BRAF mutational status. We also studied the ERK/p‐ERK levels in UT‐SCC‐42B cell line, which is a metastatic cell line originating from the same patient as UT‐SCC‐42A. In UT‐SCC‐42B, basal p‐ERK activity was even lower than in UT‐SCC‐42A cell line. Nevertheless, p‐ERK levels increased in all the cell lines after liprin‐α1 silencing, independent of the basal p‐ERK levels indicating that liprin‐α1 silencing promotes ERK signaling (Fig. 2A–D; Fig. S2A). MAPK pathway activativity score (MPAS) has been shown to be a relevant biomarker in multiple cancer types for MAPK pathway activation and MEK inhibitor sensitivity [33]. *PPFIA1* knockdown led to increased pERK levels in all our cell lines (Fig. 2D; Fig. S2B–D) while its contribution to MEK inhibitor responses was context‐dependent (Fig. S1). We thus sought to calculate MPAS from our previously published RNA‐seq and microarray data from MDA‐MB‐231, UT‐SC‐42A, and UT‐SCC‐95 cell lines [7, 9]. The MPAS value were 1.56 and 2.83 for MDA‐MB‐231 Scramble and *PPFIA1* KD cells, −3.52 and −1.3 for UT‐SCC‐42A Scramble and *PPFIA1* KD cells, and −0.83 and 1.66 for UT‐SCC‐95 *PPFIA1* OE and Ctrl cells. In all our modified cell lines, the cell line with lower *PPFIA1* expression showed higher MPAS (Fig. 2E). This result is in line with pERK levels suggesting that liprin‐α1 modulation indeed leads to MAPK pathway activation, but in our cell lines MPAS do not have better predictive power for MEKi sensitivity than pERK levels (Fig. 2E).

### 3.4 Liprin‐α1 contributes to MEK downstream signaling upon trametinib treatment in a context‐dependent manner

In *KRAS/BRAF*‐mutated MDA‐MB‐231 cells, liprin‐α1 depletion led to increased MEK/ERKi resistance while in nonmutated UT‐SCC‐42A cells *PPFIA1* silencing made cells more dependent on MEK/ERK signaling and more sensitive to MEK/ERKi treatment. To further understand the mechanisms behind the differential effect of liprin‐α1 removal to MEK/ERKi response in different cell lines, we explored the activity of the relevant signaling pathways upon treatment with trametinib, a MEK inhibitor. We thus analyzed how trametinib treatment affects ERK activation in *PPFIA1* silenced cells in three different drug concentrations. The levels of p‐ERK decreased in a concentration dependent manner in all conditions (Fig. 2F,G; Fig. S3A–D). To further understand the differential drug responses between MDA‐MB‐231 and UT‐SCC‐42A cells, we studied crosstalk between MEK‐inhibition and PI3K/AKT/mTOR activation by using phosphorylated ribosomal S6 kinase (Ser 235/236) and phosphorylated AKT (Ser473) as markers for mTORC1 and mTORC2 signaling, respectively. Trametinib treatment did not significantly decrease p‐rS6 levels after *PPFIA1* knockdown in the MDA‐MB‐231 or in the UT‐SCC‐42A cells (Fig. 2F,G). Interestingly, AKT phosphorylation at Ser473 showed a clear concentration‐dependent increase in MDA‐MB‐231 cells after trametinib treatment, while the levels remained constant in UT‐SCC‐42A cell line (Fig. 2F,G; Fig. S3A,B). This indicated that trametinib treatment affected mTORC2 activation differently in MDA‐MB‐231 and UT‐SCC‐42A cells.

### 3.5 *PPFIA1* knockdown leads to redistribution of RAS to the cell membrane and contributes to its activation

**Fig. 3:**
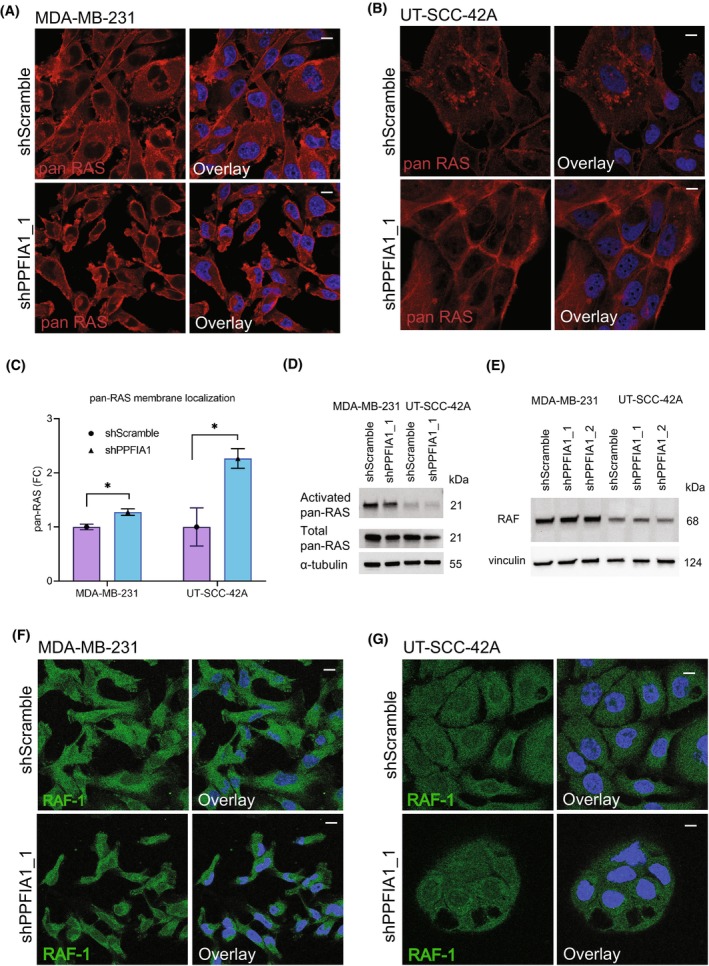
Effect of liprin‐α1 on pan‐RAS localization and RAS activity. (A) Localization of pan‐RAS in MDA‐MB‐231 shScramble and shPPFIA1 cells. Scale bar is 10 μm. Quantification of immunofluorescence staining is shown in C. (B) Localization of pan‐RAS in UT‐SCC‐42A shScramble and shPPFIA1 cells. Scale bar is 10 μm. Quantification of immunofluorescence staining is shown in C. (C) Bar plots show quantification of membrane localization of pan‐RAS in MDA‐MB‐231 (A) and UT‐SCC‐42A (B). A minimum of 70 individual cells were quantitated three times from three different experiments. Error bars have been calculated as a standard deviation of the mean. Student's two‐tailed *t*‐test was used to calculate the statistical significance. Asterisk (*) indicates statistical significance (*P* < 0.05). (D) Basal activation of Ras in cell lines with high liprin‐α1 expression (shScramble) and liprin‐α1 knockdown (shPPFIA1). Serum‐starved cells were lysed with high MgCl_2_ buffer, and the lysates were immunoprecipitated with Raf‐GST agarose beads. The precipitates were then immunoblotted with pan‐Ras antibody. Total pan‐Ras was detected from 10 μg of input lysates from each cell line and α‐tubulin was used as the loading control. Immunoblotting was carried out as a single experiment. (E) Western blot analysis of RAF in MDA‐MB‐231 and UT‐SCC‐42A cells transduced with two different knockdown constructs. Vinculin was used as the loading control. (F, G) Immunofluoresence staining of RAF‐1 in MDA‐MB‐231 (F) and in UT‐SCC‐42A shScramble and shPPFIA1 cells (G). The stainings for both of the cell lines were performed as single experiment. Scale bar is 10 μm.

RAS proteins function upstream of MEK to activate its signaling [28], which prompted us to explore whether *PPFIA1* silencing alters MEK activity via RAS. RAS can be transported to the plasma membrane, where it is activated [36]. To understand if liprin‐α1 modulates RAS activation or localization, we carried out a RAS activation assay and immunofluorescent microscopy of all RAS proteins (pan‐RAS) in control and *PPFIA1* silenced cells. In MDA‐MB‐231 cells, RAS was localized at the cell membrane in both the control and *PPFIA1* silenced cells (Fig. 3A). UT‐SCC‐42A *PPFIA1* knockdown cells showed in turn more profound RAS localization at the cell–cell contacts or cell membrane as compared to control cells (Fig. 3B,C). When studying the activation of pan‐RAS in basal conditions with overnight serum starvation, RAS was highly active in MDA‐MB‐231 as expected due to its *KRAS* mutational status (Fig. 3D). On the other hand, UT‐SCC‐42A showed only minimal basal activity of pan‐RAS compared with MDA‐MB‐231 (Fig. 3D), but activated pan‐RAS was slightly decreased upon *PPFIA1* knockdown in both cell lines (Fig. 3D). We then explored the expression of total pan‐RAS in both cell lines. In MDA‐MB‐231 and UT‐SCC‐42A cells with high *PPFIA1* expression, total pan‐RAS was highly expressed, but the level of expression decreased upon *PPFIA1* knockdown (shPPFIA1) based on immunoblotting analysis (Fig. 3D). Interestingly, liprin‐α1 depletion had no impact on RAF protein levels or localization, suggesting that contribution of liprin‐α1 signaling to pERK signaling is RAS dependent (Fig. 3E–G). The high RAS/ERK activity in MDA‐MB‐231 was in line with high p‐MEK levels (Ser217/221; Fig. 2D). Depletion of liprin‐α1 led to increase in p‐MEK levels in both cell lines, which is in line with our results on increased p‐ERK levels (Fig. 2D). As with ERK protein expression, total levels of MEK remained unchanged between conditions (Fig. 2D). These results support our previous results that liprin‐α1 contributes to MAPK signaling by counteracting RAS/MEK/ERK activity.

## 4 Discussion

*PPFIA1* encodes for liprin‐α1 protein, which contributes to the regulation of the invasive properties of cancer cells [7, 8, 9, 19, 37, 38]. *PPFIA1* is located at the 11q13 amplification region, which is associated with poor prognosis and survival of the patients [39, 40, 41, 42]. Here, we explored the potential role of liprin‐α1 as a drug response indicator, because liprin‐α1 is linked to several oncogenic processes including cancer cell invasion and adhesion [10]. We have previously shown that the impact of liprin‐α1 depletion on cancer cell invasive growth is context‐dependent [7, 9]. Therefore, we next explored the impact of liprin‐α1 depletion in drug response of targeted compounds in different cell lines with high endogenous *PPFIA1* expression. Interestingly, liprin‐α1 depletion significantly altered the response of the cells to MEK/ERKi and led to increased p‐ERK levels in all our tested cell lines. These results indicate that liprin‐α1 contributes to oncogenic signaling by regulating p‐ERK levels.

We have previously shown the association of differential cellular localization of liprin‐α1 on the invasive properties of the cells [7]. In noninvasive cells, liprin‐α1 locates to adhesive structures, such as invadosomes, while in motile cells, liprin‐α1 localizes close to the leading edge [7]. ERK controls cell motility by regulating lamellipodia formation and the rate and polarity of actin polymerization [43]. Reduced liprin‐α1 levels lead to an increase in the lamellipodia number and a decrease of their stability during migration, whereas overexpression of liprin‐α1 increases the stabilization of the lamellipodia, and the turnover of focal adhesions at the protrusive front of breast cancer cells [8, 13]. Regulation of cell migration by both liprin‐α1, as well as ERK signaling could explain the important difference in the response to MEKi upon liprin‐α1 modulation, as measured by viability and cytotoxicity.

Due to the fact that *BRAF*/*KRAS* mutation status or pERK levels are not sufficiently reliable biomarkers for MEKi sensitivity, we applied MAPK pathway activation score (MPAS), which takes into account the gene expression levels of 10 MAPK pathway genes [33]. Due to the fact that MEKi responses were variable albeit *PPFIA1* knockdown systematically led to increased pERK levels, our aim was to explore whether MPAS would provide better predictive power for MEKi sensitivity as has been shown with clinical samples [33]. In all our cell lines, MPAS was in line with pERK levels and thus, did not provide any better predictive power. MDA‐MB‐231 cells have *BRAF* mutation and constitutively active RAS/p‐ERK pathway, which drives their proliferation and survival. In tumor cells, a precise equilibrium of MEK/ERK signaling molecules is essential, and too high activity leads to either apoptosis or senescence [44]. This fact is likely to impact the large differences in DSS of MEK inhibitors between MDA‐MB‐231 scramble and liprin‐α1 depleted cells as compared to UT‐SCC cell lines, where the effect was more modest. Context‐dependency, basal p‐ERK levels, *BRAF*/*KRAS* mutation status, and differential increase in the activation of AKT between cell lines upon MEKi treatment are likely to explain the differential drug responses to a group of MEK inhibitors.

Liprin‐α1 is in complex with proteins, such as integrins, that associate with focal adhesion [7, 45]. Gain‐of‐function mutations in *RAS* genes are frequently found in human cancers [46, 47]. We show that liprin‐α1 depletion activates RAS/p‐ERK1/2 signaling by redistribution of RAS proteins to the cell membrane. In turn, liprin‐α1 does not contribute to RAF intensity or cellular localization. Opposite to MEK inhibitors, liprin‐α1 depletion causes no change in RAF inhibitors' responses between shPPFIA1 and control cells. The activation of the MAPK cascade in our studied cell lines is, thus, not bound to RAF or the intensity of RAS activation status, but is tumor type dependent and the exact mechanism remains to be elucidated. Previous data suggest a connection between liprin‐α1 and RAS signaling, as liprin‐α1 belongs to the RAS interactome in a Bir‐A proximity labeling screening [46, 47]. In addition, several scaffolding proteins have been proposed to connect ERK with signaling pathways and to target them to specific cellular compartments. Candidate scaffold proteins are identified to date, including GIT1, which interacts with liprin‐α1 to regulate cell spreading and migration [48].

## 5 Conclusions

Our study indicates that liprin‐α1 negatively regulates MEK/ERK signaling and is a potential drug response indicator in case of targeted therapeutics. Liprin‐α1 sensitizes cells to MEK/ERK inhibitors in metastatic cancer cells with constitutively active RAS, whereas cells with low endogenous p‐ERK levels show more modest change in MEKi response as compared to their liprin‐α1‐depleted counterparts. These results clearly show that while liprin‐α1 systematically counteracts ERK signaling, liprin‐α1‐dependent MEK inhibitor response is context‐dependent, although the highest response was seen in *KRAS* mutant cell line. Furthermore, liprin‐α1 depletion results in more pronounced redistribution of pan‐RAS to the cell membrane. Our results clearly demonstrate that liprin‐α1 is an important and previously unrecognized modulator of RAS/MEK/ERK signaling and a potential drug response indicator in breast and HNSCC cells which warrants further studies in clinical material.

## Conflict of interest

The authors declare no conflict of interest.

## Author contributions

HP was involved in conceptualization, formal analysis, investigation, methodology, analysis of data, data interpretation, visualization, supervision, project administration, writing—original draft, writing—editing, and review. AF was involved in conceptualization, formal analysis, investigation, methodology, analysis of data, data interpretation, visualization, writing—editing, and review. JV was involved in methodology, analysis of data, and visualization. IL was involved in methodology and analysis of data. MV and AM were involved in methodology. PI was involved in analysis of data and visualization. PM was involved in methodology and resources, JK was involved in resources, writing, and data interpretation, ST‐S was involved in resources and data interpretation. JH was involved in resources and data interpretation. RG was involved in resources, methodology, and data interpretation. AAM was involved in resources, funding acquisition, and data interpretation. P‐RK was involved in conceptualization, investigation, methodology, data interpretation, visualization, supervision, writing—editing, and review. OM was involved in conceptualization, investigation, data interpretation, resources, funding acquisition, supervision, project administration, writing—editing, and review. All the authors commented and accepted the final version of the manuscript.

## Supporting information


**Fig. S1.** Box plots showing drug group responses between *PPFIA1*‐modified and control cells. A‐D: Box plots showing differences in drug group responses between shPPFIA1 and shScramble cells or between control and *PPFIA1*‐expressing cells calculated as DSS from cell viability assay in UT‐SCC‐42A (A) and from cell viability and cytotoxicity assays in UT‐SCC‐24A, UT‐SCC‐95 and BT‐474 cell lines (B‐D).

**Fig. S2.** Effect of *PPFIA1* knockdown on p‐ERK1/2 localization and protein levels. A: p‐ERK1/2 immunostaining shows cytoplasmic localization of p‐ERK1/2 in UT‐SCC‐42A cell line. *PPFIA1* was knocked down with construct shPPFIA1_2 (#14). B: Effect of *PPFIA1* knockdown on ERK1/2 phosphorylation (Thr202/Tyr204) in UT‐SCC‐24A and BT‐474 cell lines. Two different constructs (#69 and #14) were used to knockdown *PPFIA1*. Effect of ectopic expression of *PPFIA1* on p‐ERK1/2 levels in UT‐SCC‐95 cell line. GAPDH was used as the loading control. C: Additional immunoblots demonstrating *PPFIA1* knockdown in MDA‐MB‐231 and UT‐SCC‐42A cells. D: Effect of *PPFIA1* knockdown on ERK1/2 phosphorylation (Thr202/Tyr204) in UT‐SCC‐42A and UT‐SCC‐42B HNSCC cell lines. The quantified intensities are aligned under each immunoblot.

**Fig. S3.** Effect of *PPFIA1* knockdown on downstream signaling in trametinib versus DMSO‐treated cells. A‐B: Western blot analysis of MDA‐MB‐231 (A) and UT‐SCC‐42A cells (B) treated with trametinib for 48 h. Second construct was used to knockdown *PPFIA1*. Protein levels of liprin‐α1, p‐ERK1/2 (T202/Y204), p‐AKT (Ser473) and p‐rS6 (Ser235/236) proteins are shown for shScramble and shPPFIA1 cells. DMSO‐treated cells were used as a negative control for trametinib treatment, whereas α‐tubulin and vinculin served as loading controls in immunoblotting. C‐D: Quantification of p‐ERK1/2 levels from western blots for MDA‐MB‐231 and UT‐SCC‐42A shScramble and shPPFIA1 cells treated with trametinib. DMSO was used as a negative control. Asterisk (*) shows statistical significance (p < 0.05). Four different replicates were used in C and five different replicates were used in D. Error bars were counted as a standard deviation and two‐tailed student's t‐test was used as a statistical analysis.

**Table S1.** Drug sensitivity scores (DSS) for MDA‐MB‐231, UT‐SCC‐42A, UT‐SCC‐24A, BT‐474, and UT‐SCC‐95 cell lines. A: DSS scores for all the 527 compounds tested in MDA‐MB‐231 shScramble and shPPFIA1 cells as measured by cell viability and cytotoxicity assays. B‐E: DSS scores for 23 compounds tested in UT‐SCC‐42A, UT‐SCC‐24A and BT‐474 shScramble and shPPFIA1 cells, as well as in UT‐SCC‐95 transduced with *PPFIA1*‐overexpressing and control vector.

**Tables S2–S24.** Drug response curves for MDA‐MB‐231, UT‐SCC‐42A, UT‐SCC‐24A, BT‐474, and UT‐SCC‐95 cell lines. Drug response curves for MDA‐MB‐231, UT‐SCC‐42A, UT‐SCC‐24A and BT‐474 shScramble and shPPFIA1 cells, as well as for UT‐SCC‐95 cells transduced with *PPFIA1*‐overexpressing and control vector.

## Data Availability

The microarray data [7] discussed in this publication have been deposited in The National Center for Biotechnology Information's (NCBI) Gene Expression Omnibus (GEO) and are accessible through GEO Series accession number GSE75756 (http://www.ncbi.nlm.nih.gov/geo/query/acc.cgi?&acc=GSE75756). The RNA sequencing data [9] have been deposited in NCBI's Gene Expression Omnibus and are accessible through GEO Series accession number GSE108392 (https://www.ncbi.nlm.nih.gov/geo/query/acc.cgi?acc=GSE108392). The data generated in this study are available within the article and its [Link], [Link], [Link], [Link], [Link] files and from the corresponding author upon request.
