# Supplementary figures and images for: Liprin‐α1 contributes to oncogenic MAPK signaling by counteracting ERK activity
Source: Mol Oncol. 2024 Jan 24;18(3):662–76. doi: 10.1002/1878-0261.13593 (PMC10920090; doi:10.1002/1878-0261.13593)

A.

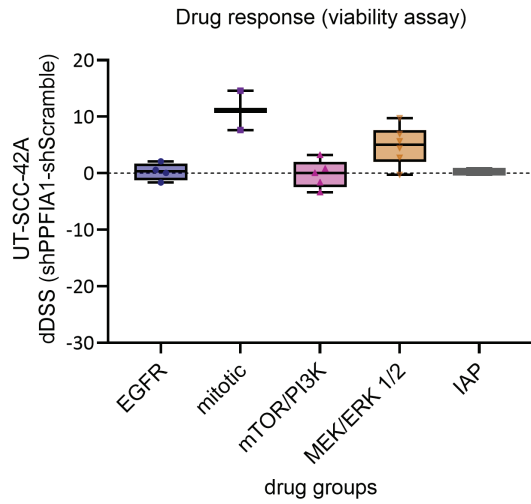

B.

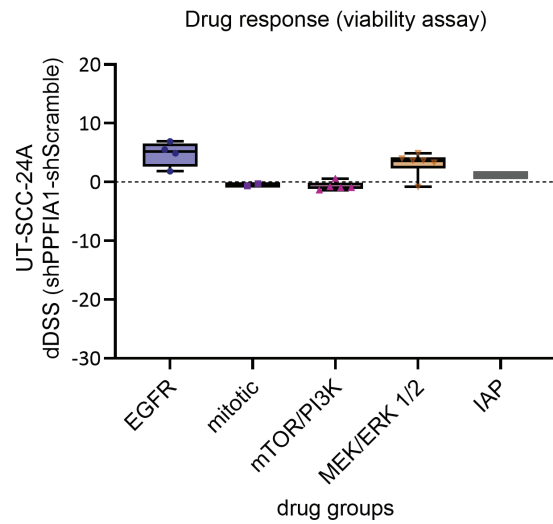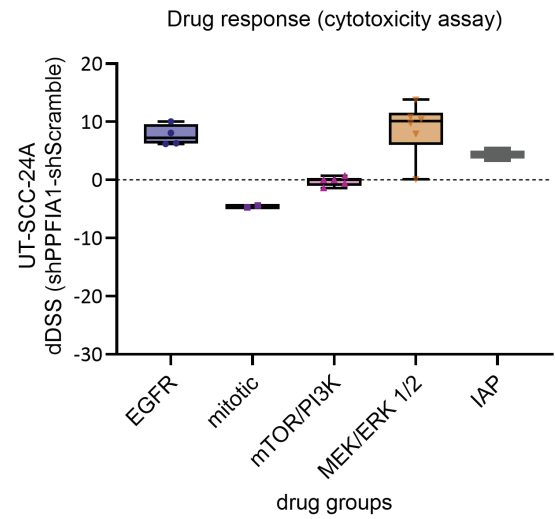

C.

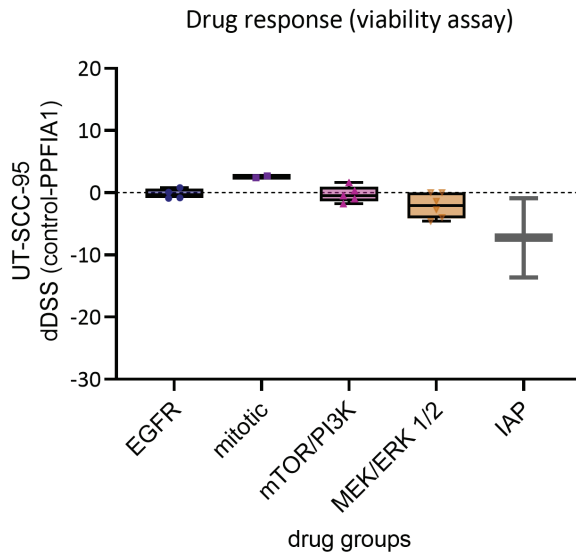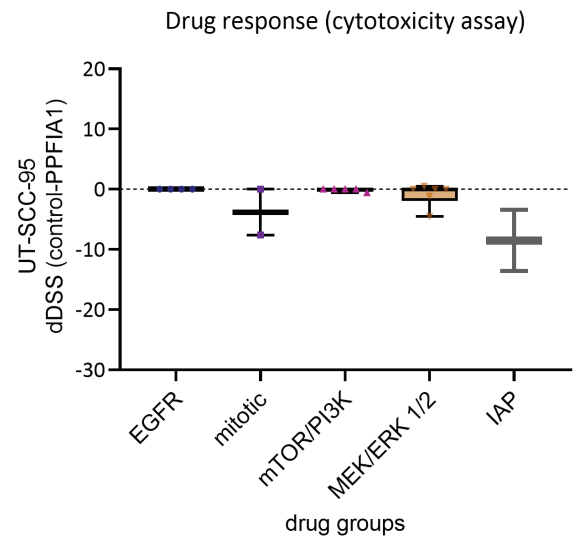

D.

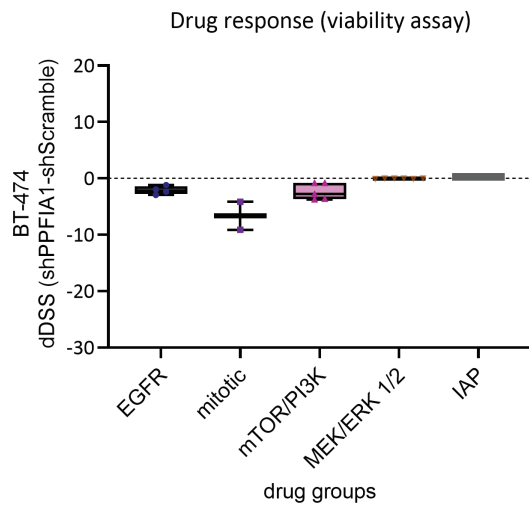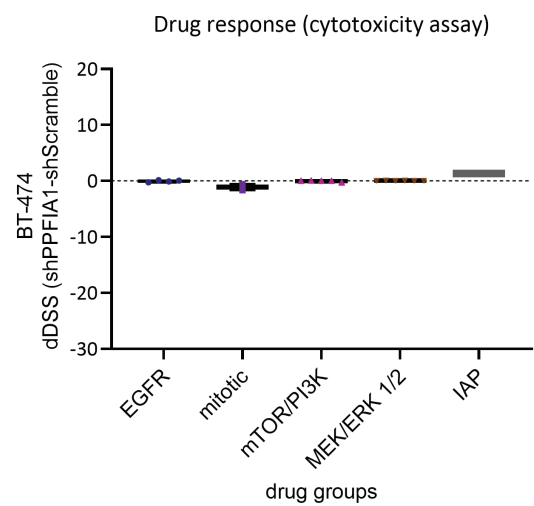

Supplement: Supplementary file 1 — Fig. S1. Box plots showing drug group responses between PPFIA1‐modified and control cells. A‐D: Box plots showing differences in drug group responses between shPPFIA1 and shScramble cells or between control and PPFIA1‐expressing cells calculated as DSS from cell viability assay in UT‐SCC‐42A (A) and from cell viability and cytotoxicity assays in UT‐SCC‐24A, UT‐SCC‐95 and BT‐474 cell lines (B‐D). [file MOL2-18-662-s005.pdf]

Supplementary Figure 2

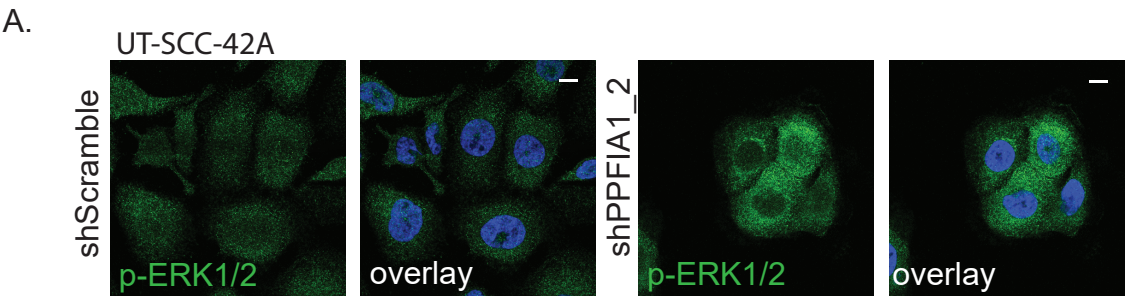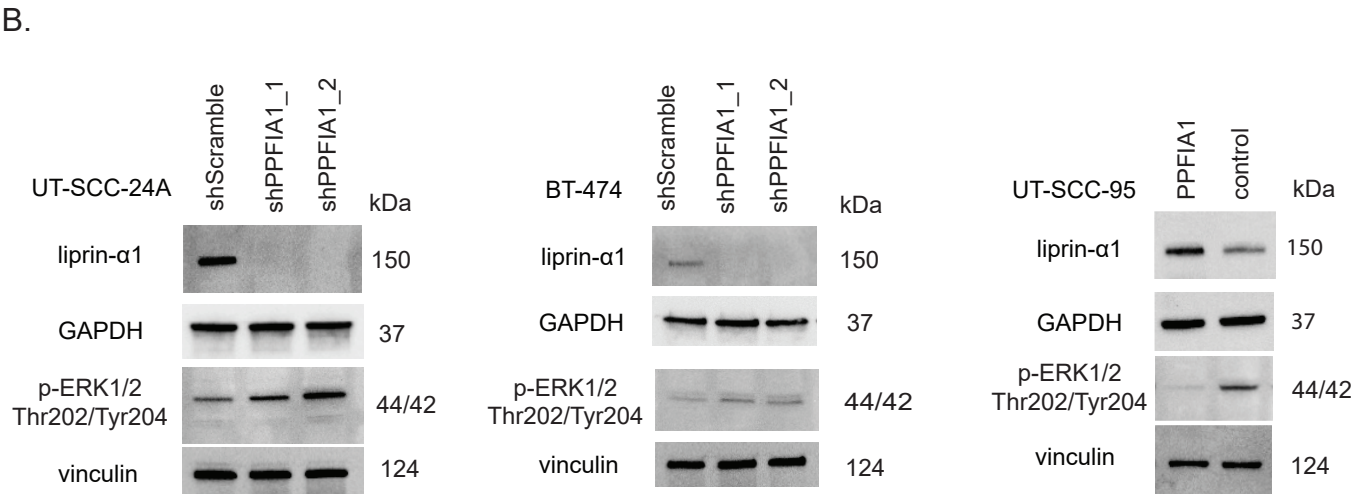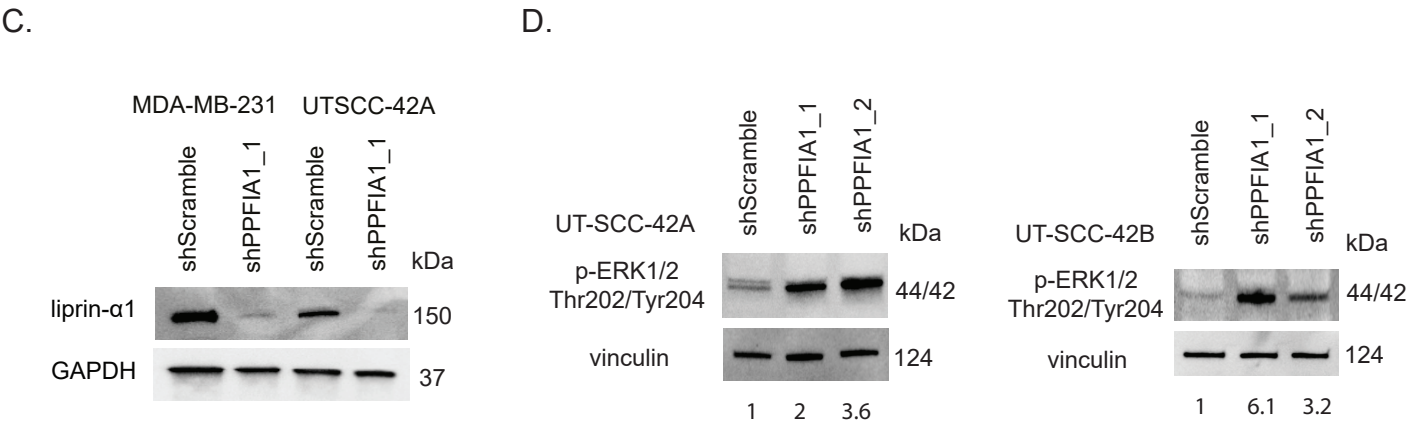

Supplement: Supplementary file 2 — Fig. S2. Effect of PPFIA1 knockdown on p‐ERK1/2 localization and protein levels. A: p‐ERK1/2 immunostaining shows cytoplasmic localization of p‐ERK1/2 in UT‐SCC‐42A cell line. PPFIA1 was knocked down with construct shPPFIA1_2 (#14). B: Effect of PPFIA1 knockdown on ERK1/2 phosphorylation (Thr202/Tyr204) in UT‐SCC‐24A and BT‐474 cell lines. Two different constructs (#69 and #14) were used to knockdown PPFIA1. Effect of ectopic expression of PPFIA1 on p‐ERK1/2 levels in UT‐SCC‐95 cell line. GAPDH was used as the loading control. C: Additional immunoblots demonstrating PPFIA1 knockdown in MDA‐MB‐231 and UT‐SCC‐42A cells. D: Effect of PPFIA1 knockdown on ERK1/2 phosphorylation (Thr202/Tyr204) in UT‐SCC‐42A and UT‐SCC‐42B HNSCC cell lines. The quantified intensities are aligned under each immunoblot. [file MOL2-18-662-s004.pdf]

Supplementary Figure 3

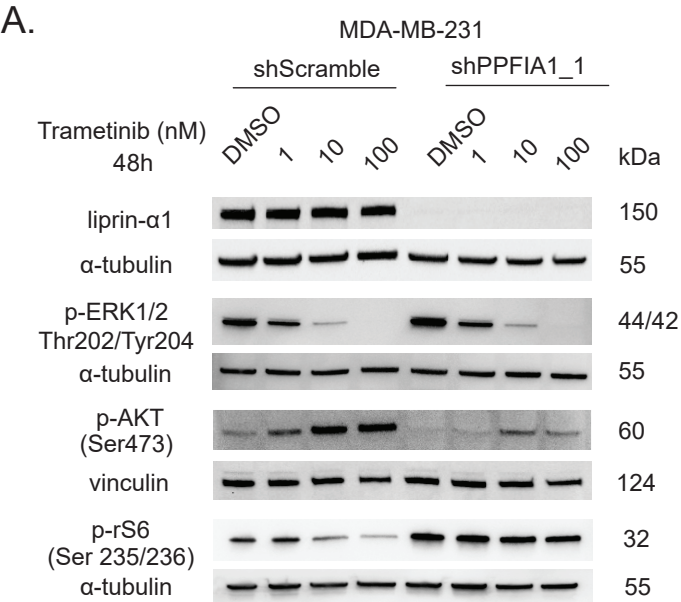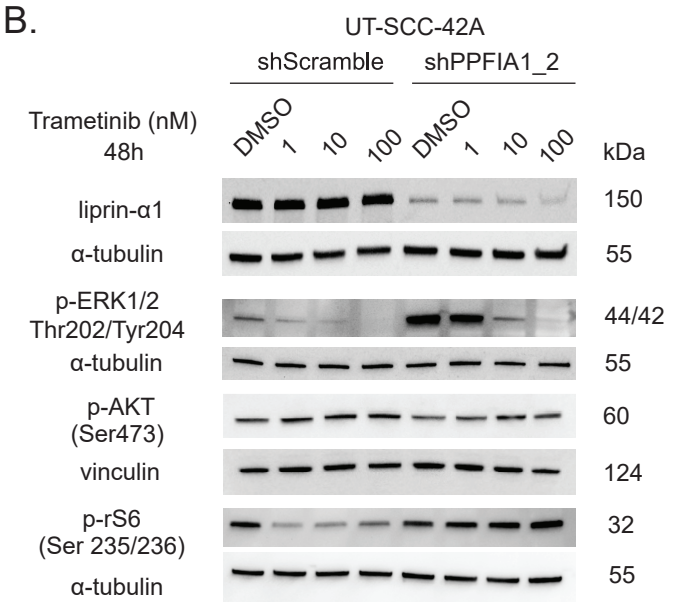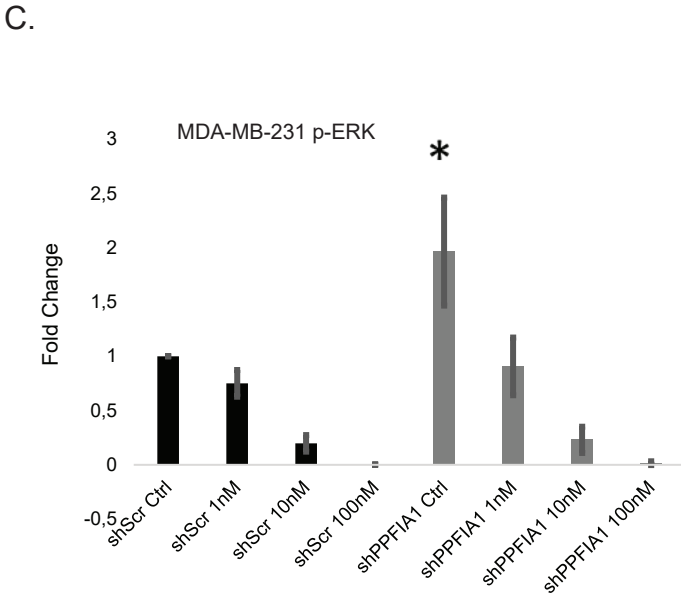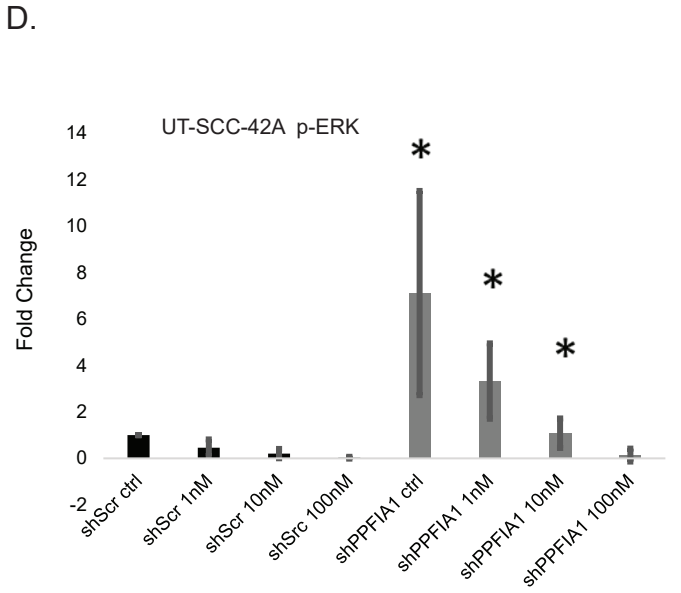

Supplement: Supplementary file 3 — Fig. S3. Effect of PPFIA1 knockdown on downstream signaling in trametinib versus DMSO‐treated cells. A‐B: Western blot analysis of MDA‐MB‐231 (A) and UT‐SCC‐42A cells (B) treated with trametinib for 48 h. Second construct was used to knockdown PPFIA1. Protein levels of liprin‐α1, p‐ERK1/2 (T202/Y204), p‐AKT (Ser473) and p‐rS6 (Ser235/236) proteins are shown for shScramble and shPPFIA1 cells. DMSO‐treated cells were used as a negative control for trametinib treatment, whereas α‐tubulin and vinculin served as loading controls in immunoblotting. C‐D: Quantification of p‐ERK1/2 levels from western blots for MDA‐MB‐231 and UT‐SCC‐42A shScramble and shPPFIA1 cells treated with trametinib. DMSO was used as a negative control. Asterisk (*) shows statistical significance (p < 0.05). Four different replicates were used in C and five different replicates were used in D. Error bars were counted as a standard deviation and two‐tailed student's t‐test was used as a statistical analysis. [file MOL2-18-662-s003.pdf]
